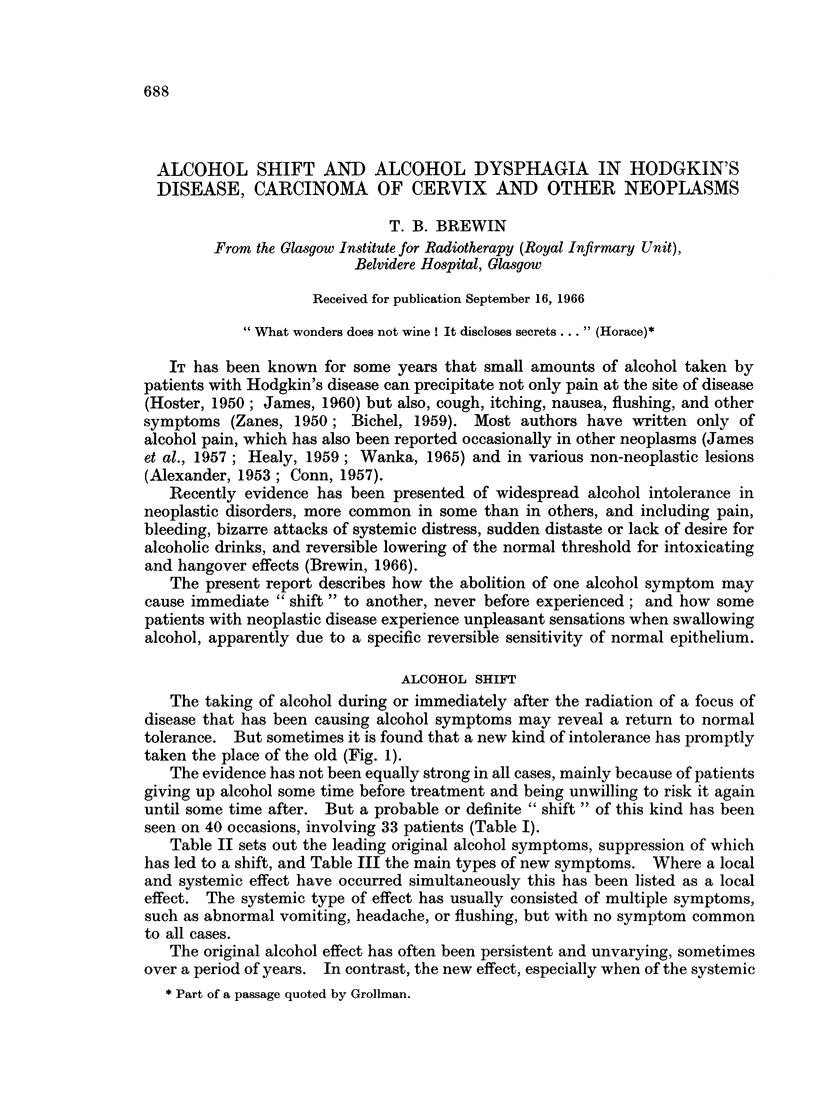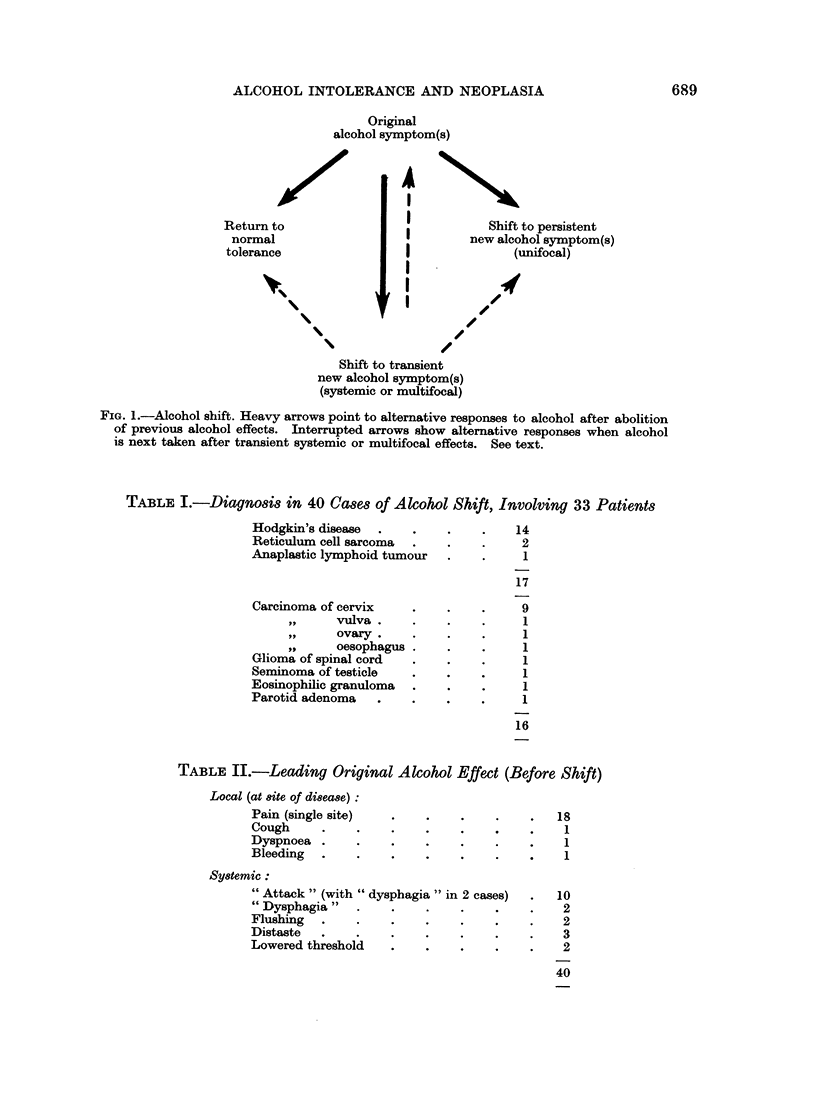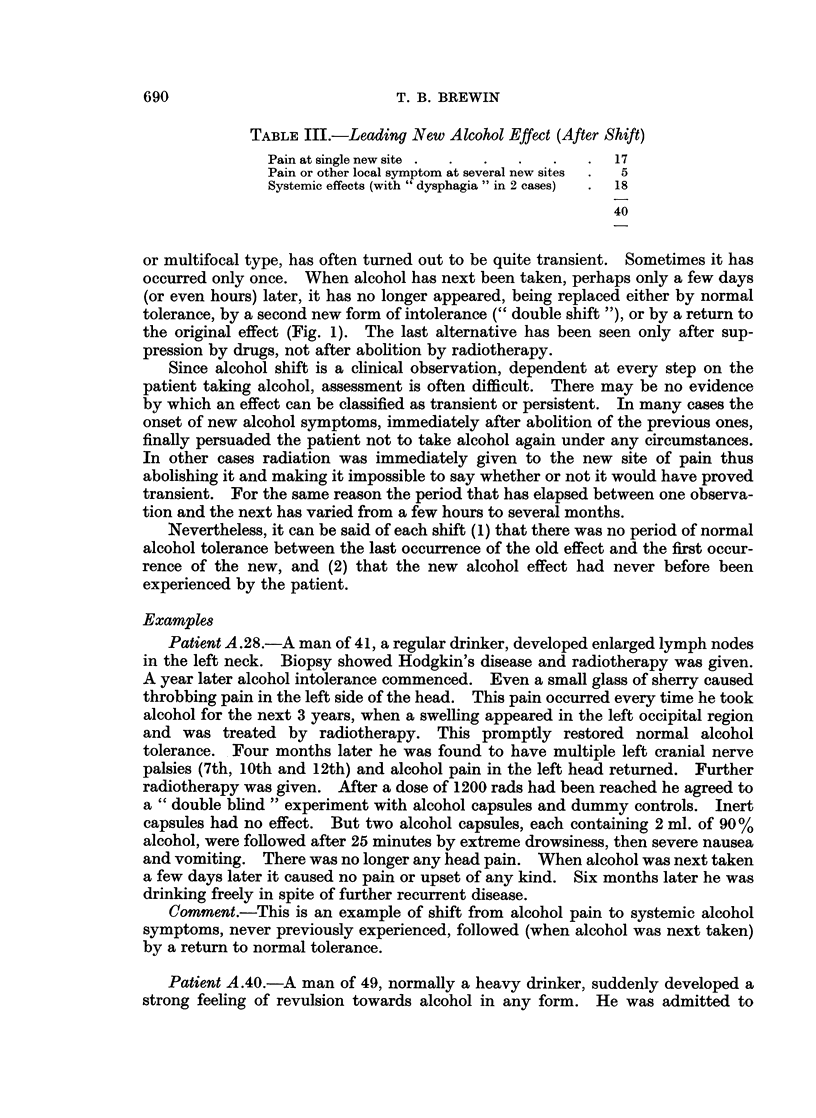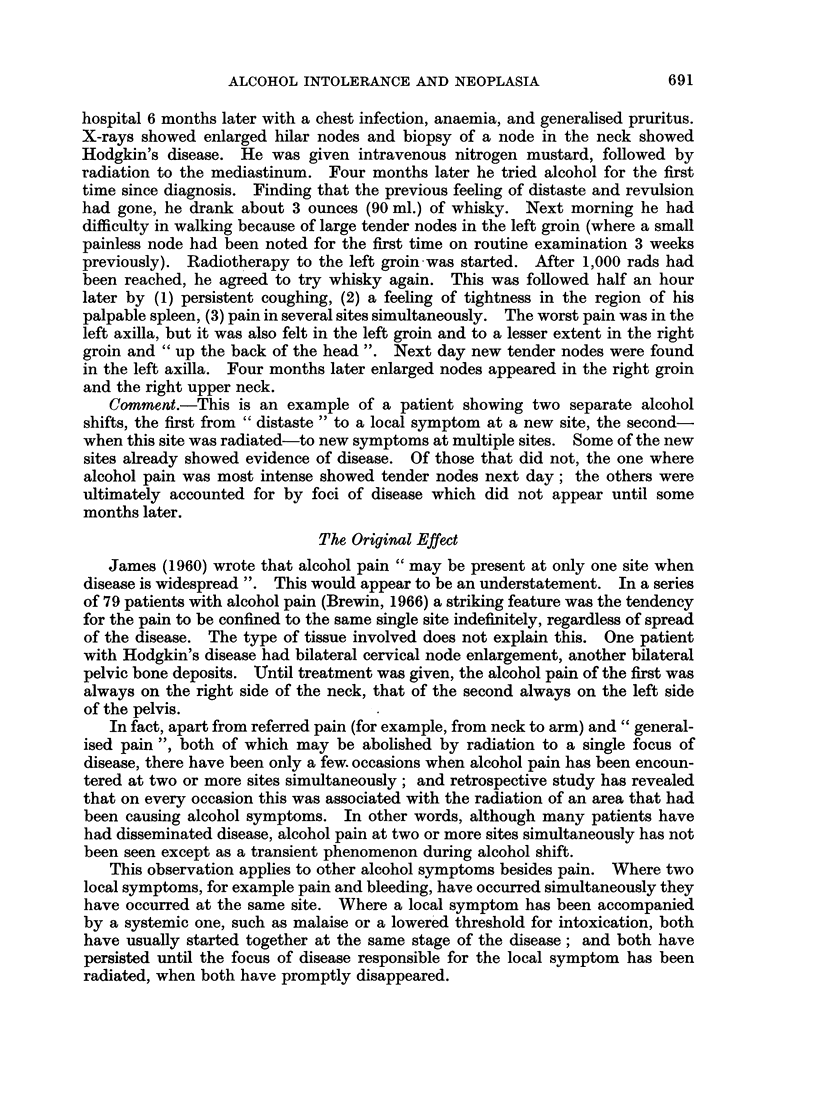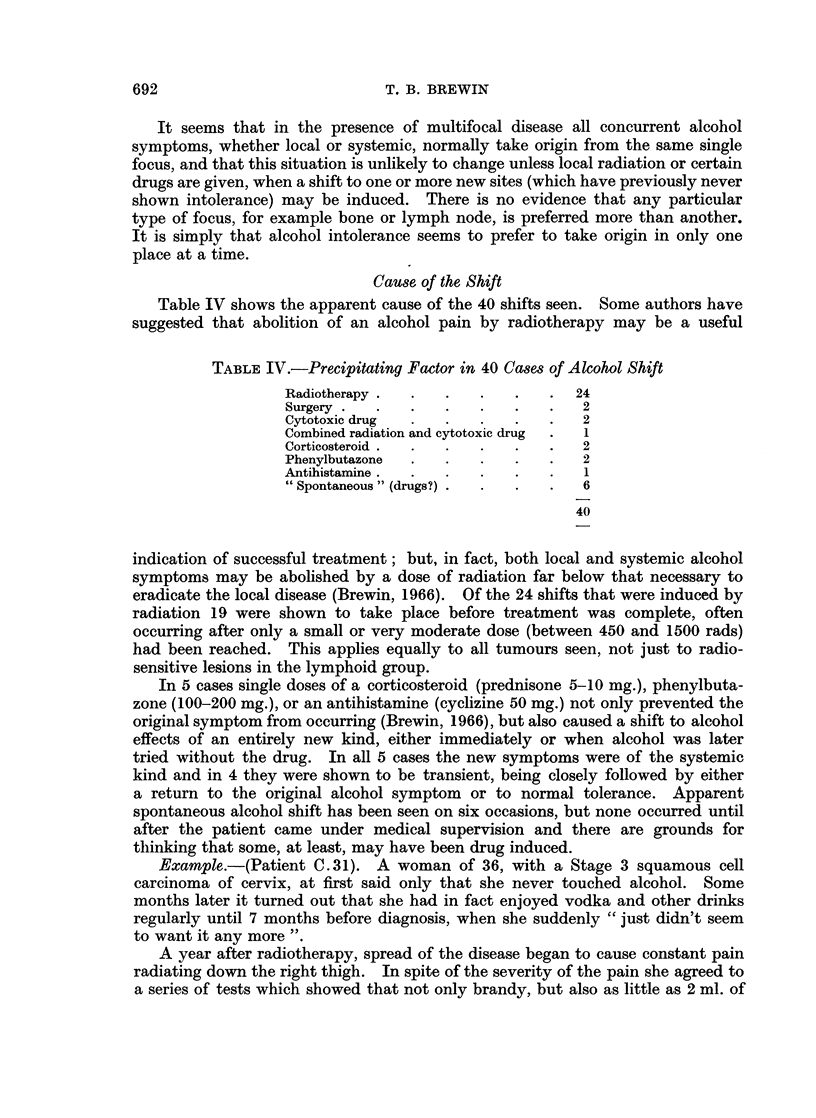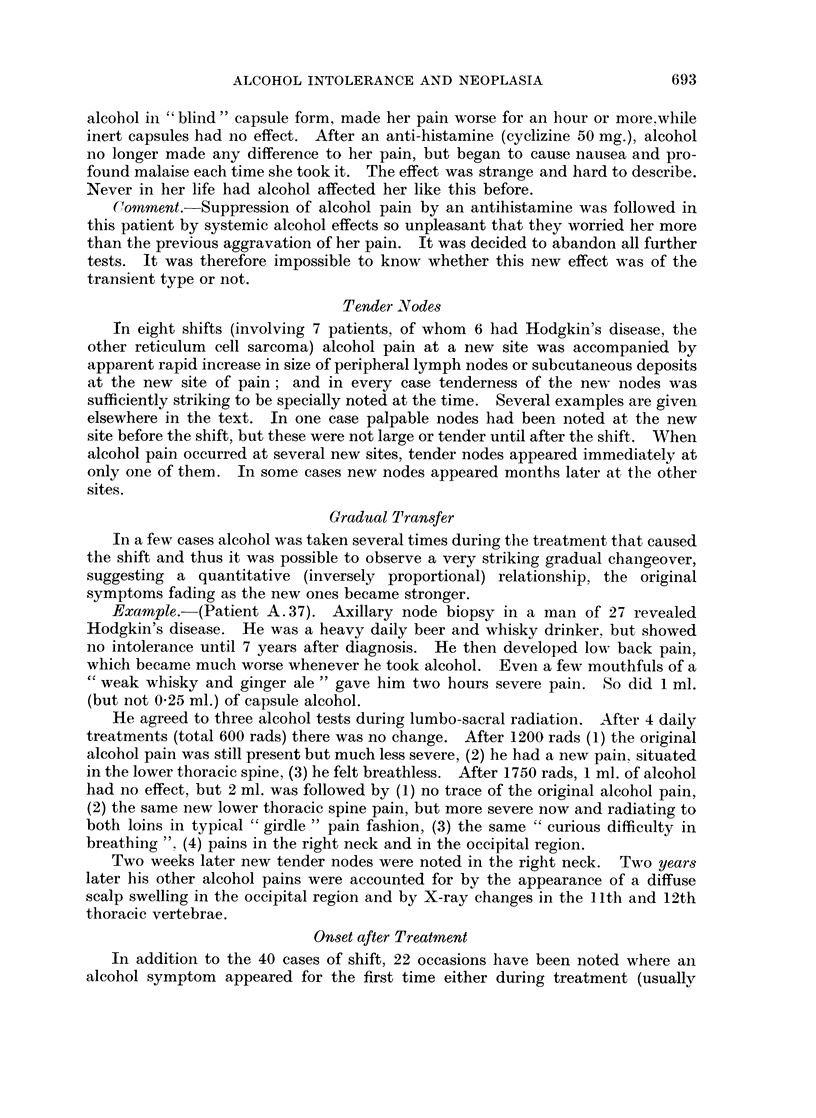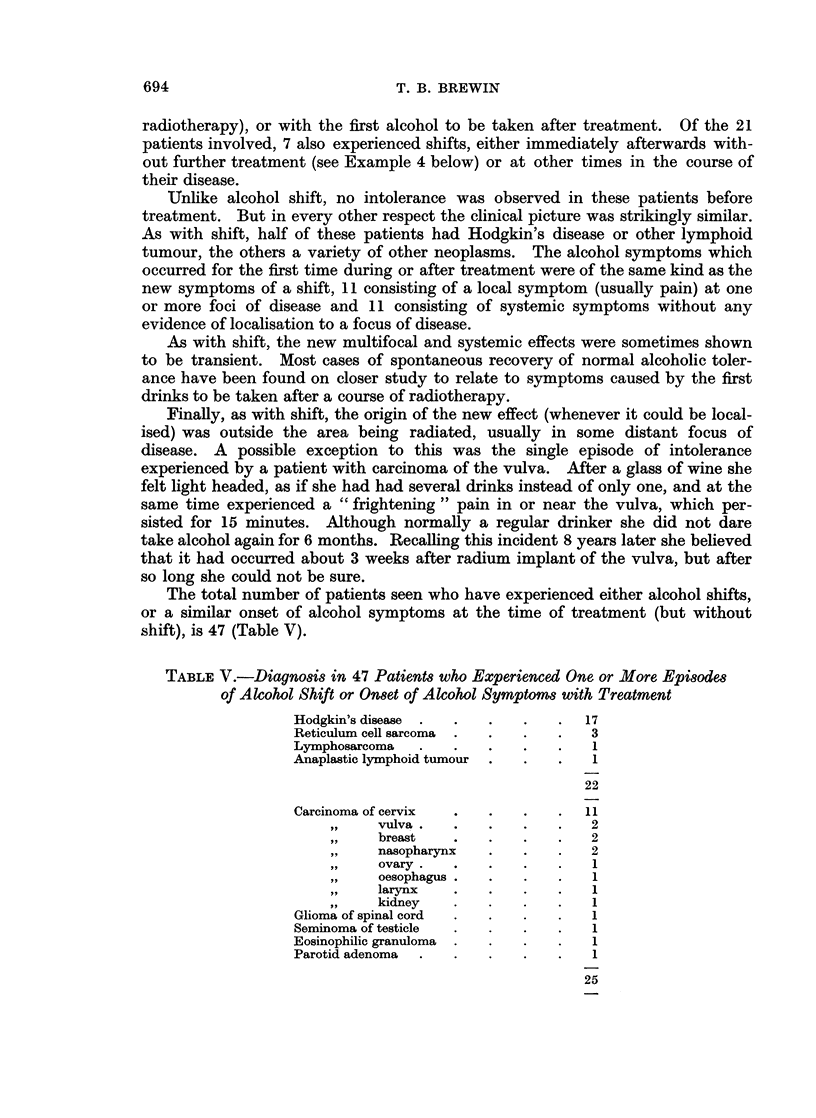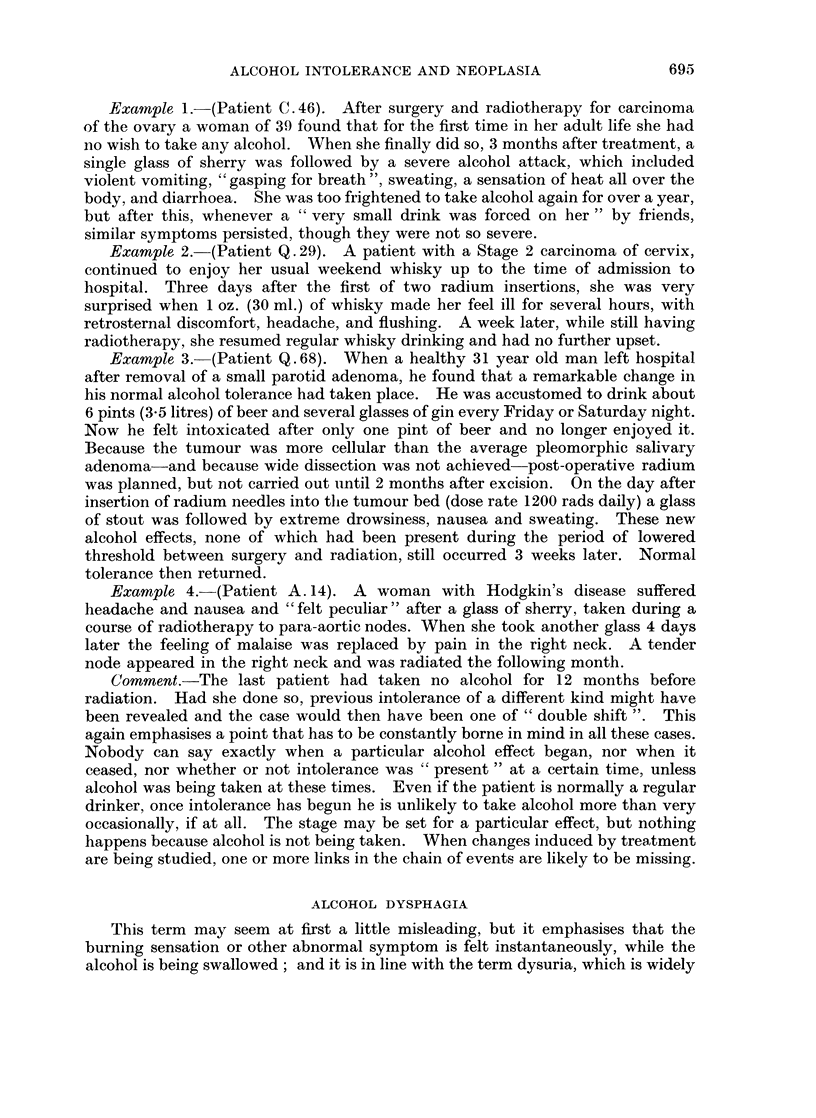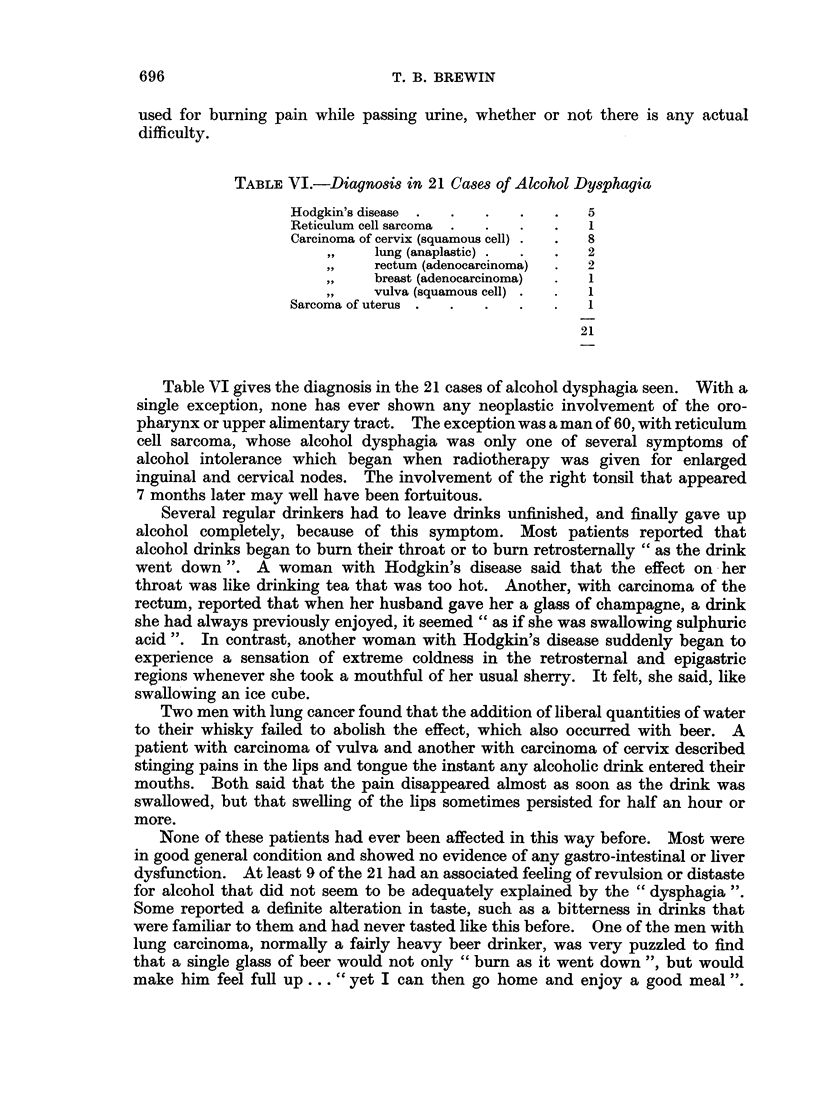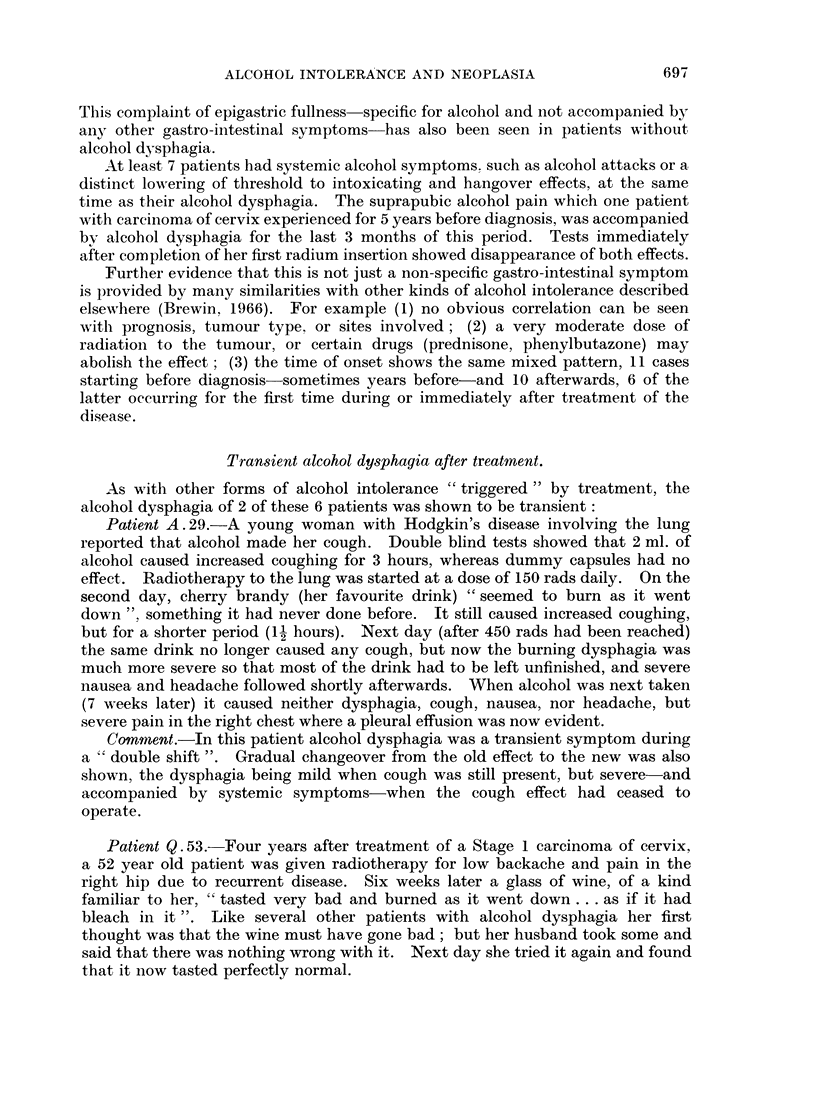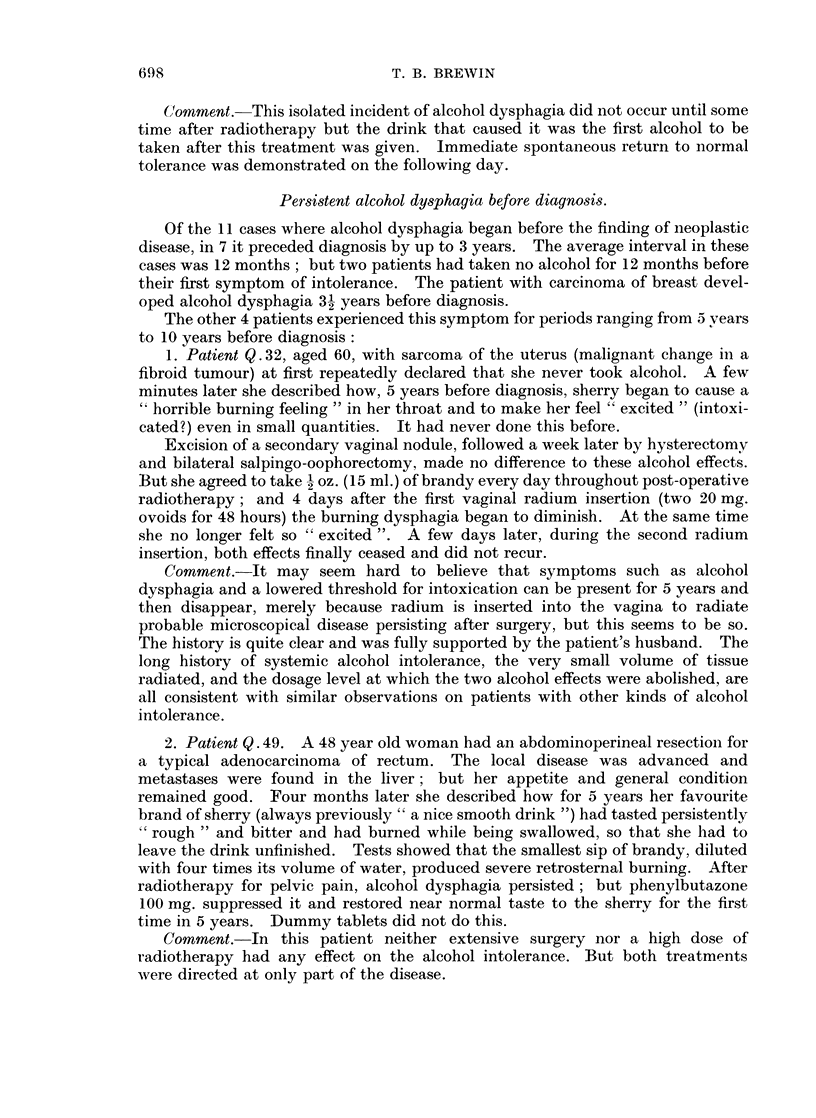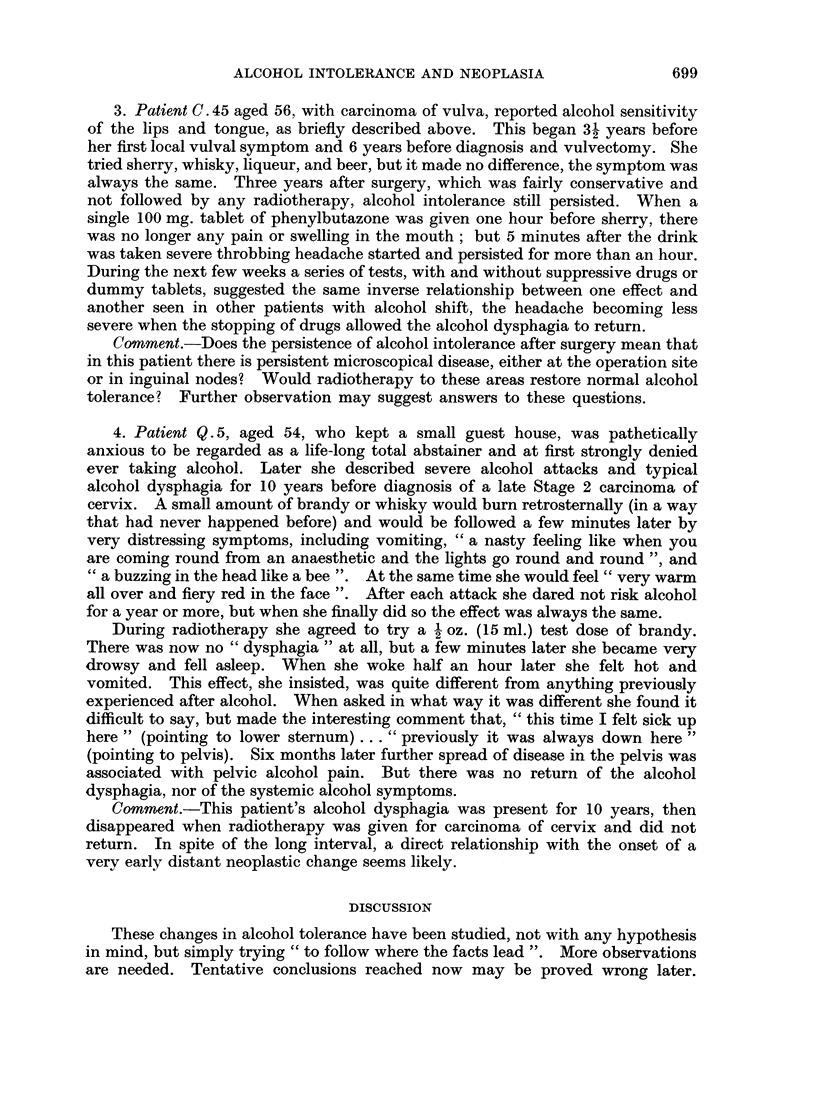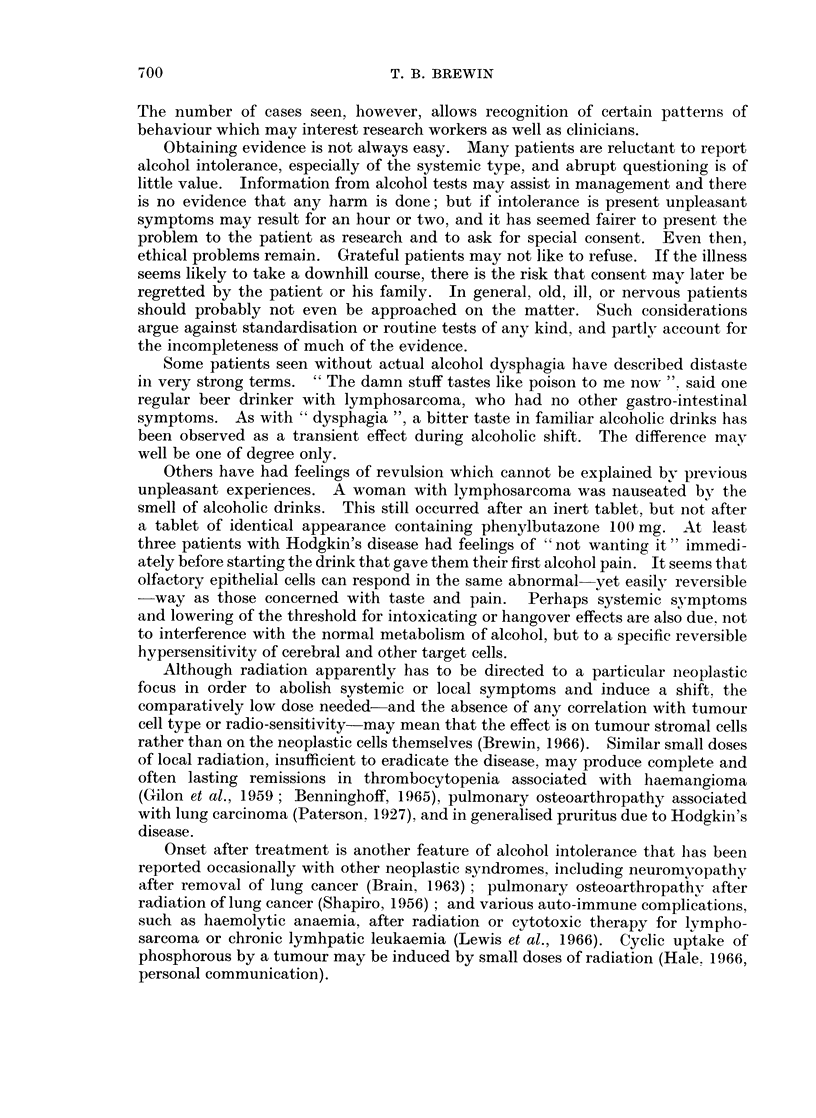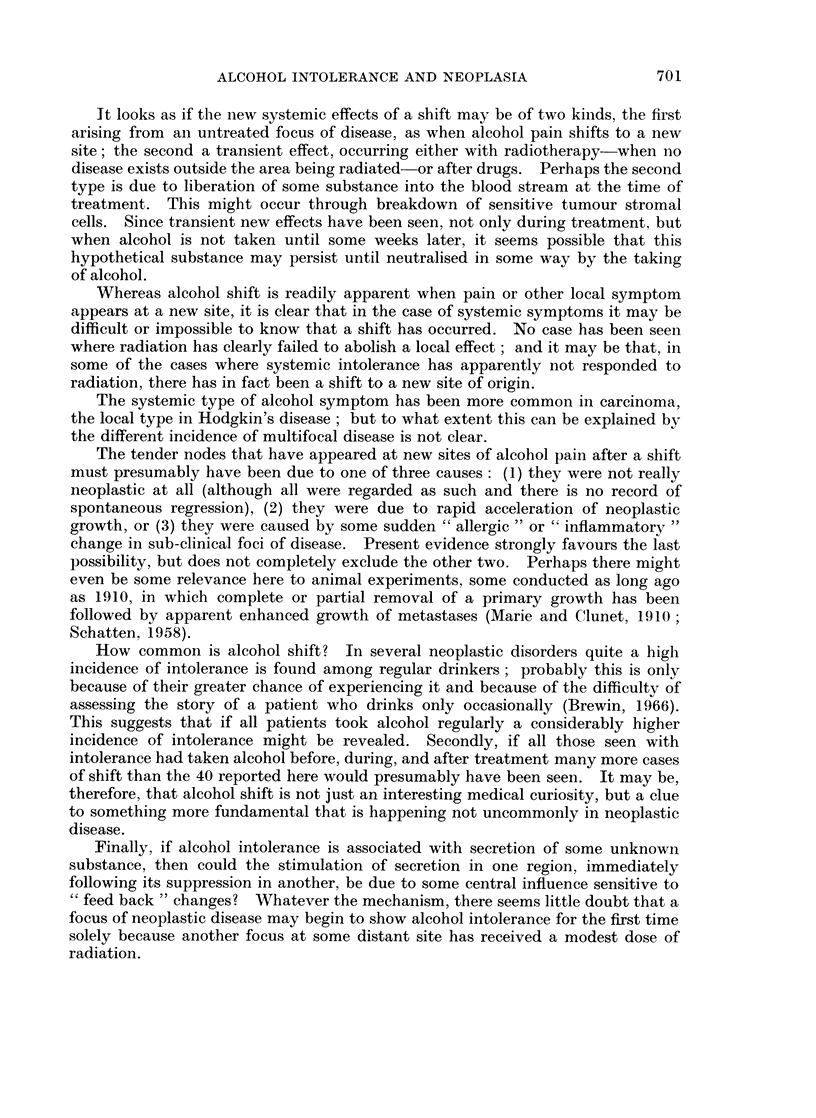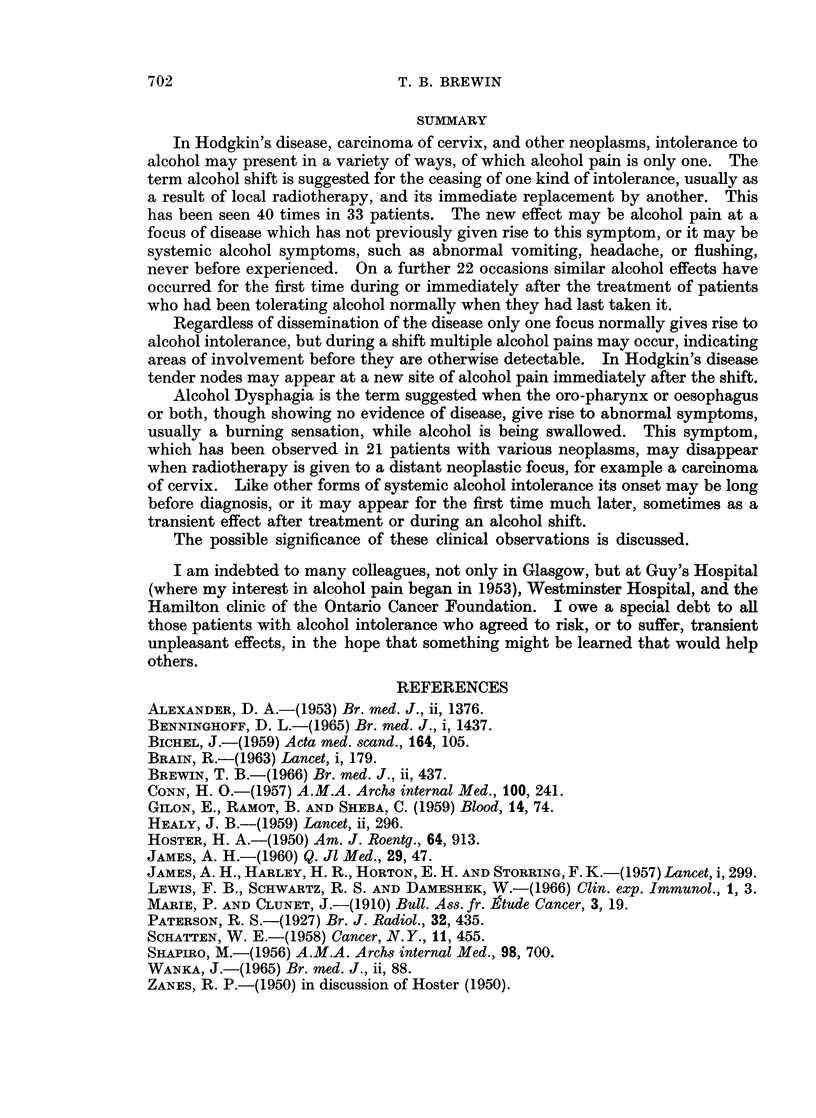# Alcohol shift and alcohol dysphagia in Hodgkin's disease, carcinoma of cervix and other neoplasms.

**DOI:** 10.1038/bjc.1966.80

**Published:** 1966-12

**Authors:** T. B. Brewin


### ALCOHOL SHIFT AND ALCOHOL DYSPHAGIA IN HODGKIN'S DISEASE, CARCINOMA OF CERVIX AND OTHER NEOPLASMS

T. B. BREWIN

*From the Glasgow Institute for Radiotherapy (Royal Infirmary Unit), Belvidere Hospital, Glasgow*

Received for publication September 16, 1966

" What wonders does not wine ! It discloses secrets . . . " (Horace)*

IT has been known for some years that small amounts of alcohol taken by patients with Hodgkin's disease can precipitate not only pain at the site of disease (Hoster, 1950 ; James, 1960) but also, cough, itching, nausea, flushing, and other symptoms (Zanes, 1950 ; Bichel, 1959). Most authors have written only of alcohol pain, which has also been reported occasionally in other neoplasms (James *et al.*, 1957 ; Healy, 1959 ; Wanka, 1965) and in various non-neoplastic lesions (Alexander, 1953 ; Conn, 1957).

Recently evidence has been presented of widespread alcohol intolerance in neoplastic disorders, more common in some than in others, and including pain, bleeding, bizarre attacks of systemic distress, sudden distaste or lack of desire for alcoholic drinks, and reversible lowering of the normal threshold for intoxicating and hangover effects (Brewin, 1966).

The present report describes how the abolition of one alcohol symptom may cause immediate " shift " to another, never before experienced ; and how some patients with neoplastic disease experience unpleasant sensations when swallowing alcohol, apparently due to a specific reversible sensitivity of normal epithelium.

### ALCOHOL SHIFT

The taking of alcohol during or immediately after the radiation of a focus of disease that has been causing alcohol symptoms may reveal a return to normal tolerance. But sometimes it is found that a new kind of intolerance has promptly taken the place of the old (Fig. 1).

The evidence has not been equally strong in all cases, mainly because of patients giving up alcohol some time before treatment and being unwilling to risk it again until some time after. But a probable or definite " shift " of this kind has been seen on 40 occasions, involving 33 patients (Table I).

Table II sets out the leading original alcohol symptoms, suppression of which has led to a shift, and Table III the main types of new symptoms. Where a local and systemic effect have occurred simultaneously this has been listed as a local effect. The systemic type of effect has usually consisted of multiple symptoms, such as abnormal vomiting, headache, or flushing, but with no symptom common to all cases.

The original alcohol effect has often been persistent and unvarying, sometimes over a period of years. In contrast, the new effect, especially when of the systemic

* Part of a passage quoted by Grollman.

FIG. 1.—Alcohol shift. Heavy arrows point to alternative responses to alcohol after abolition of previous alcohol effects. Interrupted arrows show alternative responses when alcohol is next taken after transient systemic or multifocal effects. See text.

TABLE I.—*Diagnosis in* 40 *Cases of Alcohol Shift, Involving* 33 *Patients*

| | |
|---|---|
| Hodgkin's disease . . . . | 14 |
| Reticulum cell sarcoma . . . | 2 |
| Anaplastic lymphoid tumour . . | 1 |
| | 17 |
| Carcinoma of cervix . . . | 9 |
| „ vulva . . . . | 1 |
| „ ovary . . . . | 1 |
| „ oesophagus . . . | 1 |
| Glioma of spinal cord . . . | 1 |
| Seminoma of testicle . . . | 1 |
| Eosinophilic granuloma . . . | 1 |
| Parotid adenoma . . . . | 1 |
| | 16 |

TABLE II.—*Leading Original Alcohol Effect* (*Before Shift*)

| | |
|---|---|
| *Local* (*at site of disease*) *:* | |
| Pain (single site) . . . . . . | 18 |
| Cough . . . . . . . . | 1 |
| Dyspnoea . . . . . . . . | 1 |
| Bleeding . . . . . . . . | 1 |
| *Systemic :* | |
| " Attack " (with " dysphagia " in 2 cases) . | 10 |
| " Dysphagia " . . . . . . . | 2 |
| Flushing . . . . . . . . | 2 |
| Distaste . . . . . . . . | 3 |
| Lowered threshold . . . . . . | 2 |
| | 40 |

TABLE III.—*Leading New Alcohol Effect (After Shift)*

| | |
|---|---|
| Pain at single new site . . . . . . . | 17 |
| Pain or other local symptom at several new sites . | 5 |
| Systemic effects (with " dysphagia " in 2 cases) . | 18 |
| | 40 |

or multifocal type, has often turned out to be quite transient. Sometimes it has occurred only once. When alcohol has next been taken, perhaps only a few days (or even hours) later, it has no longer appeared, being replaced either by normal tolerance, by a second new form of intolerance (" double shift "), or by a return to the original effect (Fig. 1). The last alternative has been seen only after suppression by drugs, not after abolition by radiotherapy.

Since alcohol shift is a clinical observation, dependent at every step on the patient taking alcohol, assessment is often difficult. There may be no evidence by which an effect can be classified as transient or persistent. In many cases the onset of new alcohol symptoms, immediately after abolition of the previous ones, finally persuaded the patient not to take alcohol again under any circumstances. In other cases radiation was immediately given to the new site of pain thus abolishing it and making it impossible to say whether or not it would have proved transient. For the same reason the period that has elapsed between one observation and the next has varied from a few hours to several months.

Nevertheless, it can be said of each shift (1) that there was no period of normal alcohol tolerance between the last occurrence of the old effect and the first occurrence of the new, and (2) that the new alcohol effect had never before been experienced by the patient.

*Examples*

*Patient A*.28.—A man of 41, a regular drinker, developed enlarged lymph nodes in the left neck. Biopsy showed Hodgkin's disease and radiotherapy was given. A year later alcohol intolerance commenced. Even a small glass of sherry caused throbbing pain in the left side of the head. This pain occurred every time he took alcohol for the next 3 years, when a swelling appeared in the left occipital region and was treated by radiotherapy. This promptly restored normal alcohol tolerance. Four months later he was found to have multiple left cranial nerve palsies (7th, 10th and 12th) and alcohol pain in the left head returned. Further radiotherapy was given. After a dose of 1200 rads had been reached he agreed to a " double blind " experiment with alcohol capsules and dummy controls. Inert capsules had no effect. But two alcohol capsules, each containing 2 ml. of 90% alcohol, were followed after 25 minutes by extreme drowsiness, then severe nausea and vomiting. There was no longer any head pain. When alcohol was next taken a few days later it caused no pain or upset of any kind. Six months later he was drinking freely in spite of further recurrent disease.

*Comment.*—This is an example of shift from alcohol pain to systemic alcohol symptoms, never previously experienced, followed (when alcohol was next taken) by a return to normal tolerance.

*Patient A*.40.—A man of 49, normally a heavy drinker, suddenly developed a strong feeling of revulsion towards alcohol in any form. He was admitted to hospital 6 months later with a chest infection, anaemia, and generalised pruritus. X-rays showed enlarged hilar nodes and biopsy of a node in the neck showed Hodgkin's disease. He was given intravenous nitrogen mustard, followed by radiation to the mediastinum. Four months later he tried alcohol for the first time since diagnosis. Finding that the previous feeling of distaste and revulsion had gone, he drank about 3 ounces (90 ml.) of whisky. Next morning he had difficulty in walking because of large tender nodes in the left groin (where a small painless node had been noted for the first time on routine examination 3 weeks previously). Radiotherapy to the left groin was started. After 1,000 rads had been reached, he agreed to try whisky again. This was followed half an hour later by (1) persistent coughing, (2) a feeling of tightness in the region of his palpable spleen, (3) pain in several sites simultaneously. The worst pain was in the left axilla, but it was also felt in the left groin and to a lesser extent in the right groin and " up the back of the head ". Next day new tender nodes were found in the left axilla. Four months later enlarged nodes appeared in the right groin and the right upper neck.

*Comment.*—This is an example of a patient showing two separate alcohol shifts, the first from " distaste " to a local symptom at a new site, the second—when this site was radiated—to new symptoms at multiple sites. Some of the new sites already showed evidence of disease. Of those that did not, the one where alcohol pain was most intense showed tender nodes next day ; the others were ultimately accounted for by foci of disease which did not appear until some months later.

### *The Original Effect*

James (1960) wrote that alcohol pain " may be present at only one site when disease is widespread ". This would appear to be an understatement. In a series of 79 patients with alcohol pain (Brewin, 1966) a striking feature was the tendency for the pain to be confined to the same single site indefinitely, regardless of spread of the disease. The type of tissue involved does not explain this. One patient with Hodgkin's disease had bilateral cervical node enlargement, another bilateral pelvic bone deposits. Until treatment was given, the alcohol pain of the first was always on the right side of the neck, that of the second always on the left side of the pelvis.

In fact, apart from referred pain (for example, from neck to arm) and " generalised pain ", both of which may be abolished by radiation to a single focus of disease, there have been only a few occasions when alcohol pain has been encountered at two or more sites simultaneously ; and retrospective study has revealed that on every occasion this was associated with the radiation of an area that had been causing alcohol symptoms. In other words, although many patients have had disseminated disease, alcohol pain at two or more sites simultaneously has not been seen except as a transient phenomenon during alcohol shift.

This observation applies to other alcohol symptoms besides pain. Where two local symptoms, for example pain and bleeding, have occurred simultaneously they have occurred at the same site. Where a local symptom has been accompanied by a systemic one, such as malaise or a lowered threshold for intoxication, both have usually started together at the same stage of the disease ; and both have persisted until the focus of disease responsible for the local symptom has been radiated, when both have promptly disappeared.

It seems that in the presence of multifocal disease all concurrent alcohol symptoms, whether local or systemic, normally take origin from the same single focus, and that this situation is unlikely to change unless local radiation or certain drugs are given, when a shift to one or more new sites (which have previously never shown intolerance) may be induced. There is no evidence that any particular type of focus, for example bone or lymph node, is preferred more than another. It is simply that alcohol intolerance seems to prefer to take origin in only one place at a time.

*Cause of the Shift*

Table IV shows the apparent cause of the 40 shifts seen. Some authors have suggested that abolition of an alcohol pain by radiotherapy may be a useful

TABLE IV.—*Precipitating Factor in* 40 *Cases of Alcohol Shift*

| | |
|---|---|
| Radiotherapy | 24 |
| Surgery | 2 |
| Cytotoxic drug | 2 |
| Combined radiation and cytotoxic drug | 1 |
| Corticosteroid | 2 |
| Phenylbutazone | 2 |
| Antihistamine | 1 |
| " Spontaneous " (drugs?) | 6 |
| | 40 |

indication of successful treatment; but, in fact, both local and systemic alcohol symptoms may be abolished by a dose of radiation far below that necessary to eradicate the local disease (Brewin, 1966). Of the 24 shifts that were induced by radiation 19 were shown to take place before treatment was complete, often occurring after only a small or very moderate dose (between 450 and 1500 rads) had been reached. This applies equally to all tumours seen, not just to radiosensitive lesions in the lymphoid group.

In 5 cases single doses of a corticosteroid (prednisone 5–10 mg.), phenylbutazone (100–200 mg.), or an antihistamine (cyclizine 50 mg.) not only prevented the original symptom from occurring (Brewin, 1966), but also caused a shift to alcohol effects of an entirely new kind, either immediately or when alcohol was later tried without the drug. In all 5 cases the new symptoms were of the systemic kind and in 4 they were shown to be transient, being closely followed by either a return to the original alcohol symptom or to normal tolerance. Apparent spontaneous alcohol shift has been seen on six occasions, but none occurred until after the patient came under medical supervision and there are grounds for thinking that some, at least, may have been drug induced.

*Example.*—(Patient C.31). A woman of 36, with a Stage 3 squamous cell carcinoma of cervix, at first said only that she never touched alcohol. Some months later it turned out that she had in fact enjoyed vodka and other drinks regularly until 7 months before diagnosis, when she suddenly " just didn't seem to want it any more ".

A year after radiotherapy, spread of the disease began to cause constant pain radiating down the right thigh. In spite of the severity of the pain she agreed to a series of tests which showed that not only brandy, but also as little as 2 ml. of alcohol in "blind" capsule form, made her pain worse for an hour or more, while inert capsules had no effect. After an anti-histamine (cyclizine 50 mg.), alcohol no longer made any difference to her pain, but began to cause nausea and profound malaise each time she took it. The effect was strange and hard to describe. Never in her life had alcohol affected her like this before.

*Comment.*—Suppression of alcohol pain by an antihistamine was followed in this patient by systemic alcohol effects so unpleasant that they worried her more than the previous aggravation of her pain. It was decided to abandon all further tests. It was therefore impossible to know whether this new effect was of the transient type or not.

### *Tender Nodes*

In eight shifts (involving 7 patients, of whom 6 had Hodgkin's disease, the other reticulum cell sarcoma) alcohol pain at a new site was accompanied by apparent rapid increase in size of peripheral lymph nodes or subcutaneous deposits at the new site of pain; and in every case tenderness of the new nodes was sufficiently striking to be specially noted at the time. Several examples are given elsewhere in the text. In one case palpable nodes had been noted at the new site before the shift, but these were not large or tender until after the shift. When alcohol pain occurred at several new sites, tender nodes appeared immediately at only one of them. In some cases new nodes appeared months later at the other sites.

### *Gradual Transfer*

In a few cases alcohol was taken several times during the treatment that caused the shift and thus it was possible to observe a very striking gradual changeover, suggesting a quantitative (inversely proportional) relationship, the original symptoms fading as the new ones became stronger.

*Example.*—(Patient A.37). Axillary node biopsy in a man of 27 revealed Hodgkin's disease. He was a heavy daily beer and whisky drinker, but showed no intolerance until 7 years after diagnosis. He then developed low back pain, which became much worse whenever he took alcohol. Even a few mouthfuls of a "weak whisky and ginger ale" gave him two hours severe pain. So did 1 ml. (but not 0·25 ml.) of capsule alcohol.

He agreed to three alcohol tests during lumbo-sacral radiation. After 4 daily treatments (total 600 rads) there was no change. After 1200 rads (1) the original alcohol pain was still present but much less severe, (2) he had a new pain, situated in the lower thoracic spine, (3) he felt breathless. After 1750 rads, 1 ml. of alcohol had no effect, but 2 ml. was followed by (1) no trace of the original alcohol pain, (2) the same new lower thoracic spine pain, but more severe now and radiating to both loins in typical "girdle" pain fashion, (3) the same "curious difficulty in breathing", (4) pains in the right neck and in the occipital region.

Two weeks later new tender nodes were noted in the right neck. Two *years* later his other alcohol pains were accounted for by the appearance of a diffuse scalp swelling in the occipital region and by X-ray changes in the 11th and 12th thoracic vertebrae.

### *Onset after Treatment*

In addition to the 40 cases of shift, 22 occasions have been noted where an alcohol symptom appeared for the first time either during treatment (usually radiotherapy), or with the first alcohol to be taken after treatment. Of the 21 patients involved, 7 also experienced shifts, either immediately afterwards without further treatment (see Example 4 below) or at other times in the course of their disease.

Unlike alcohol shift, no intolerance was observed in these patients before treatment. But in every other respect the clinical picture was strikingly similar. As with shift, half of these patients had Hodgkin's disease or other lymphoid tumour, the others a variety of other neoplasms. The alcohol symptoms which occurred for the first time during or after treatment were of the same kind as the new symptoms of a shift, 11 consisting of a local symptom (usually pain) at one or more foci of disease and 11 consisting of systemic symptoms without any evidence of localisation to a focus of disease.

As with shift, the new multifocal and systemic effects were sometimes shown to be transient. Most cases of spontaneous recovery of normal alcoholic tolerance have been found on closer study to relate to symptoms caused by the first drinks to be taken after a course of radiotherapy.

Finally, as with shift, the origin of the new effect (whenever it could be localised) was outside the area being radiated, usually in some distant focus of disease. A possible exception to this was the single episode of intolerance experienced by a patient with carcinoma of the vulva. After a glass of wine she felt light headed, as if she had had several drinks instead of only one, and at the same time experienced a "frightening" pain in or near the vulva, which persisted for 15 minutes. Although normally a regular drinker she did not dare take alcohol again for 6 months. Recalling this incident 8 years later she believed that it had occurred about 3 weeks after radium implant of the vulva, but after so long she could not be sure.

The total number of patients seen who have experienced either alcohol shifts, or a similar onset of alcohol symptoms at the time of treatment (but without shift), is 47 (Table V).

TABLE V.—*Diagnosis in 47 Patients who Experienced One or More Episodes of Alcohol Shift or Onset of Alcohol Symptoms with Treatment*

| | |
|---|---|
| Hodgkin's disease | 17 |
| Reticulum cell sarcoma | 3 |
| Lymphosarcoma | 1 |
| Anaplastic lymphoid tumour | 1 |
| | 22 |
| Carcinoma of cervix | 11 |
| ,, vulva | 2 |
| ,, breast | 2 |
| ,, nasopharynx | 2 |
| ,, ovary | 1 |
| ,, oesophagus | 1 |
| ,, larynx | 1 |
| ,, kidney | 1 |
| Glioma of spinal cord | 1 |
| Seminoma of testicle | 1 |
| Eosinophilic granuloma | 1 |
| Parotid adenoma | 1 |
| | 25 |

*Example* 1.—(Patient C.46). After surgery and radiotherapy for carcinoma of the ovary a woman of 39 found that for the first time in her adult life she had no wish to take any alcohol. When she finally did so, 3 months after treatment, a single glass of sherry was followed by a severe alcohol attack, which included violent vomiting, "gasping for breath", sweating, a sensation of heat all over the body, and diarrhoea. She was too frightened to take alcohol again for over a year, but after this, whenever a "very small drink was forced on her" by friends, similar symptoms persisted, though they were not so severe.

*Example* 2.—(Patient Q.29). A patient with a Stage 2 carcinoma of cervix, continued to enjoy her usual weekend whisky up to the time of admission to hospital. Three days after the first of two radium insertions, she was very surprised when 1 oz. (30 ml.) of whisky made her feel ill for several hours, with retrosternal discomfort, headache, and flushing. A week later, while still having radiotherapy, she resumed regular whisky drinking and had no further upset.

*Example* 3.—(Patient Q.68). When a healthy 31 year old man left hospital after removal of a small parotid adenoma, he found that a remarkable change in his normal alcohol tolerance had taken place. He was accustomed to drink about 6 pints (3·5 litres) of beer and several glasses of gin every Friday or Saturday night. Now he felt intoxicated after only one pint of beer and no longer enjoyed it. Because the tumour was more cellular than the average pleomorphic salivary adenoma—and because wide dissection was not achieved—post-operative radium was planned, but not carried out until 2 months after excision. On the day after insertion of radium needles into the tumour bed (dose rate 1200 rads daily) a glass of stout was followed by extreme drowsiness, nausea and sweating. These new alcohol effects, none of which had been present during the period of lowered threshold between surgery and radiation, still occurred 3 weeks later. Normal tolerance then returned.

*Example* 4.—(Patient A.14). A woman with Hodgkin's disease suffered headache and nausea and "felt peculiar" after a glass of sherry, taken during a course of radiotherapy to para-aortic nodes. When she took another glass 4 days later the feeling of malaise was replaced by pain in the right neck. A tender node appeared in the right neck and was radiated the following month.

*Comment.*—The last patient had taken no alcohol for 12 months before radiation. Had she done so, previous intolerance of a different kind might have been revealed and the case would then have been one of "double shift". This again emphasises a point that has to be constantly borne in mind in all these cases. Nobody can say exactly when a particular alcohol effect began, nor when it ceased, nor whether or not intolerance was "present" at a certain time, unless alcohol was being taken at these times. Even if the patient is normally a regular drinker, once intolerance has begun he is unlikely to take alcohol more than very occasionally, if at all. The stage may be set for a particular effect, but nothing happens because alcohol is not being taken. When changes induced by treatment are being studied, one or more links in the chain of events are likely to be missing.

### ALCOHOL DYSPHAGIA

This term may seem at first a little misleading, but it emphasises that the burning sensation or other abnormal symptom is felt instantaneously, while the alcohol is being swallowed ; and it is in line with the term dysuria, which is widely used for burning pain while passing urine, whether or not there is any actual difficulty.

TABLE VI.—*Diagnosis in 21 Cases of Alcohol Dysphagia*

| | |
|---|---|
| Hodgkin's disease | 5 |
| Reticulum cell sarcoma | 1 |
| Carcinoma of cervix (squamous cell) | 8 |
| ,, lung (anaplastic) | 2 |
| ,, rectum (adenocarcinoma) | 2 |
| ,, breast (adenocarcinoma) | 1 |
| ,, vulva (squamous cell) | 1 |
| Sarcoma of uterus | 1 |
| | 21 |

Table VI gives the diagnosis in the 21 cases of alcohol dysphagia seen. With a single exception, none has ever shown any neoplastic involvement of the oro-pharynx or upper alimentary tract. The exception was a man of 60, with reticulum cell sarcoma, whose alcohol dysphagia was only one of several symptoms of alcohol intolerance which began when radiotherapy was given for enlarged inguinal and cervical nodes. The involvement of the right tonsil that appeared 7 months later may well have been fortuitous.

Several regular drinkers had to leave drinks unfinished, and finally gave up alcohol completely, because of this symptom. Most patients reported that alcohol drinks began to burn their throat or to burn retrosternally " as the drink went down ". A woman with Hodgkin's disease said that the effect on her throat was like drinking tea that was too hot. Another, with carcinoma of the rectum, reported that when her husband gave her a glass of champagne, a drink she had always previously enjoyed, it seemed " as if she was swallowing sulphuric acid ". In contrast, another woman with Hodgkin's disease suddenly began to experience a sensation of extreme coldness in the retrosternal and epigastric regions whenever she took a mouthful of her usual sherry. It felt, she said, like swallowing an ice cube.

Two men with lung cancer found that the addition of liberal quantities of water to their whisky failed to abolish the effect, which also occurred with beer. A patient with carcinoma of vulva and another with carcinoma of cervix described stinging pains in the lips and tongue the instant any alcoholic drink entered their mouths. Both said that the pain disappeared almost as soon as the drink was swallowed, but that swelling of the lips sometimes persisted for half an hour or more.

None of these patients had ever been affected in this way before. Most were in good general condition and showed no evidence of any gastro-intestinal or liver dysfunction. At least 9 of the 21 had an associated feeling of revulsion or distaste for alcohol that did not seem to be adequately explained by the " dysphagia ". Some reported a definite alteration in taste, such as a bitterness in drinks that were familiar to them and had never tasted like this before. One of the men with lung carcinoma, normally a fairly heavy beer drinker, was very puzzled to find that a single glass of beer would not only " burn as it went down ", but would make him feel full up . . . " yet I can then go home and enjoy a good meal ".

This complaint of epigastric fullness—specific for alcohol and not accompanied by any other gastro-intestinal symptoms—has also been seen in patients without alcohol dysphagia.

At least 7 patients had systemic alcohol symptoms, such as alcohol attacks or a distinct lowering of threshold to intoxicating and hangover effects, at the same time as their alcohol dysphagia. The suprapubic alcohol pain which one patient with carcinoma of cervix experienced for 5 years before diagnosis, was accompanied by alcohol dysphagia for the last 3 months of this period. Tests immediately after completion of her first radium insertion showed disappearance of both effects.

Further evidence that this is not just a non-specific gastro-intestinal symptom is provided by many similarities with other kinds of alcohol intolerance described elsewhere (Brewin, 1966). For example (1) no obvious correlation can be seen with prognosis, tumour type, or sites involved ; (2) a very moderate dose of radiation to the tumour, or certain drugs (prednisone, phenylbutazone) may abolish the effect ; (3) the time of onset shows the same mixed pattern, 11 cases starting before diagnosis—sometimes years before—and 10 afterwards, 6 of the latter occurring for the first time during or immediately after treatment of the disease.

*Transient alcohol dysphagia after treatment.*

As with other forms of alcohol intolerance " triggered " by treatment, the alcohol dysphagia of 2 of these 6 patients was shown to be transient :

*Patient A*.29.—A young woman with Hodgkin's disease involving the lung reported that alcohol made her cough. Double blind tests showed that 2 ml. of alcohol caused increased coughing for 3 hours, whereas dummy capsules had no effect. Radiotherapy to the lung was started at a dose of 150 rads daily. On the second day, cherry brandy (her favourite drink) " seemed to burn as it went down ", something it had never done before. It still caused increased coughing, but for a shorter period ($1\frac{1}{2}$ hours). Next day (after 450 rads had been reached) the same drink no longer caused any cough, but now the burning dysphagia was much more severe so that most of the drink had to be left unfinished, and severe nausea and headache followed shortly afterwards. When alcohol was next taken (7 weeks later) it caused neither dysphagia, cough, nausea, nor headache, but severe pain in the right chest where a pleural effusion was now evident.

*Comment.*—In this patient alcohol dysphagia was a transient symptom during a " double shift ". Gradual changeover from the old effect to the new was also shown, the dysphagia being mild when cough was still present, but severe—and accompanied by systemic symptoms—when the cough effect had ceased to operate.

*Patient Q*.53.—Four years after treatment of a Stage 1 carcinoma of cervix, a 52 year old patient was given radiotherapy for low backache and pain in the right hip due to recurrent disease. Six weeks later a glass of wine, of a kind familiar to her, " tasted very bad and burned as it went down . . . as if it had bleach in it ". Like several other patients with alcohol dysphagia her first thought was that the wine must have gone bad ; but her husband took some and said that there was nothing wrong with it. Next day she tried it again and found that it now tasted perfectly normal.

*Comment*.—This isolated incident of alcohol dysphagia did not occur until some time after radiotherapy but the drink that caused it was the first alcohol to be taken after this treatment was given. Immediate spontaneous return to normal tolerance was demonstrated on the following day.

*Persistent alcohol dysphagia before diagnosis.*

Of the 11 cases where alcohol dysphagia began before the finding of neoplastic disease, in 7 it preceded diagnosis by up to 3 years. The average interval in these cases was 12 months ; but two patients had taken no alcohol for 12 months before their first symptom of intolerance. The patient with carcinoma of breast developed alcohol dysphagia $3\frac{1}{2}$ years before diagnosis.

The other 4 patients experienced this symptom for periods ranging from 5 years to 10 years before diagnosis :

1. *Patient Q*.32, aged 60, with sarcoma of the uterus (malignant change in a fibroid tumour) at first repeatedly declared that she never took alcohol. A few minutes later she described how, 5 years before diagnosis, sherry began to cause a " horrible burning feeling " in her throat and to make her feel " excited " (intoxicated?) even in small quantities. It had never done this before.

Excision of a secondary vaginal nodule, followed a week later by hysterectomy and bilateral salpingo-oophorectomy, made no difference to these alcohol effects. But she agreed to take $\frac{1}{2}$ oz. (15 ml.) of brandy every day throughout post-operative radiotherapy ; and 4 days after the first vaginal radium insertion (two 20 mg. ovoids for 48 hours) the burning dysphagia began to diminish. At the same time she no longer felt so " excited ". A few days later, during the second radium insertion, both effects finally ceased and did not recur.

*Comment*.—It may seem hard to believe that symptoms such as alcohol dysphagia and a lowered threshold for intoxication can be present for 5 years and then disappear, merely because radium is inserted into the vagina to radiate probable microscopical disease persisting after surgery, but this seems to be so. The history is quite clear and was fully supported by the patient's husband. The long history of systemic alcohol intolerance, the very small volume of tissue radiated, and the dosage level at which the two alcohol effects were abolished, are all consistent with similar observations on patients with other kinds of alcohol intolerance.

2. *Patient Q*.49. A 48 year old woman had an abdominoperineal resection for a typical adenocarcinoma of rectum. The local disease was advanced and metastases were found in the liver ; but her appetite and general condition remained good. Four months later she described how for 5 years her favourite brand of sherry (always previously " a nice smooth drink ") had tasted persistently " rough " and bitter and had burned while being swallowed, so that she had to leave the drink unfinished. Tests showed that the smallest sip of brandy, diluted with four times its volume of water, produced severe retrosternal burning. After radiotherapy for pelvic pain, alcohol dysphagia persisted ; but phenylbutazone 100 mg. suppressed it and restored near normal taste to the sherry for the first time in 5 years. Dummy tablets did not do this.

*Comment*.—In this patient neither extensive surgery nor a high dose of radiotherapy had any effect on the alcohol intolerance. But both treatments were directed at only part of the disease.

3. *Patient C.*45 aged 56, with carcinoma of vulva, reported alcohol sensitivity of the lips and tongue, as briefly described above. This began $3\frac{1}{2}$ years before her first local vulval symptom and 6 years before diagnosis and vulvectomy. She tried sherry, whisky, liqueur, and beer, but it made no difference, the symptom was always the same. Three years after surgery, which was fairly conservative and not followed by any radiotherapy, alcohol intolerance still persisted. When a single 100 mg. tablet of phenylbutazone was given one hour before sherry, there was no longer any pain or swelling in the mouth ; but 5 minutes after the drink was taken severe throbbing headache started and persisted for more than an hour. During the next few weeks a series of tests, with and without suppressive drugs or dummy tablets, suggested the same inverse relationship between one effect and another seen in other patients with alcohol shift, the headache becoming less severe when the stopping of drugs allowed the alcohol dysphagia to return.

*Comment.*—Does the persistence of alcohol intolerance after surgery mean that in this patient there is persistent microscopical disease, either at the operation site or in inguinal nodes? Would radiotherapy to these areas restore normal alcohol tolerance? Further observation may suggest answers to these questions.

4. *Patient Q.*5, aged 54, who kept a small guest house, was pathetically anxious to be regarded as a life-long total abstainer and at first strongly denied ever taking alcohol. Later she described severe alcohol attacks and typical alcohol dysphagia for 10 years before diagnosis of a late Stage 2 carcinoma of cervix. A small amount of brandy or whisky would burn retrosternally (in a way that had never happened before) and would be followed a few minutes later by very distressing symptoms, including vomiting, " a nasty feeling like when you are coming round from an anaesthetic and the lights go round and round ", and " a buzzing in the head like a bee ". At the same time she would feel " very warm all over and fiery red in the face ". After each attack she dared not risk alcohol for a year or more, but when she finally did so the effect was always the same.

During radiotherapy she agreed to try a $\frac{1}{2}$ oz. (15 ml.) test dose of brandy. There was now no " dysphagia " at all, but a few minutes later she became very drowsy and fell asleep. When she woke half an hour later she felt hot and vomited. This effect, she insisted, was quite different from anything previously experienced after alcohol. When asked in what way it was different she found it difficult to say, but made the interesting comment that, " this time I felt sick up here " (pointing to lower sternum) . . . " previously it was always down here " (pointing to pelvis). Six months later further spread of disease in the pelvis was associated with pelvic alcohol pain. But there was no return of the alcohol dysphagia, nor of the systemic alcohol symptoms.

*Comment.*—This patient's alcohol dysphagia was present for 10 years, then disappeared when radiotherapy was given for carcinoma of cervix and did not return. In spite of the long interval, a direct relationship with the onset of a very early distant neoplastic change seems likely.

### DISCUSSION

These changes in alcohol tolerance have been studied, not with any hypothesis in mind, but simply trying " to follow where the facts lead ". More observations are needed. Tentative conclusions reached now may be proved wrong later.

The number of cases seen, however, allows recognition of certain patterns of behaviour which may interest research workers as well as clinicians.

Obtaining evidence is not always easy. Many patients are reluctant to report alcohol intolerance, especially of the systemic type, and abrupt questioning is of little value. Information from alcohol tests may assist in management and there is no evidence that any harm is done; but if intolerance is present unpleasant symptoms may result for an hour or two, and it has seemed fairer to present the problem to the patient as research and to ask for special consent. Even then, ethical problems remain. Grateful patients may not like to refuse. If the illness seems likely to take a downhill course, there is the risk that consent may later be regretted by the patient or his family. In general, old, ill, or nervous patients should probably not even be approached on the matter. Such considerations argue against standardisation or routine tests of any kind, and partly account for the incompleteness of much of the evidence.

Some patients seen without actual alcohol dysphagia have described distaste in very strong terms. " The damn stuff tastes like poison to me now ", said one regular beer drinker with lymphosarcoma, who had no other gastro-intestinal symptoms. As with " dysphagia ", a bitter taste in familiar alcoholic drinks has been observed as a transient effect during alcoholic shift. The difference may well be one of degree only.

Others have had feelings of revulsion which cannot be explained by previous unpleasant experiences. A woman with lymphosarcoma was nauseated by the smell of alcoholic drinks. This still occurred after an inert tablet, but not after a tablet of identical appearance containing phenylbutazone 100 mg. At least three patients with Hodgkin's disease had feelings of "not wanting it" immediately before starting the drink that gave them their first alcohol pain. It seems that olfactory epithelial cells can respond in the same abnormal—yet easily reversible—way as those concerned with taste and pain. Perhaps systemic symptoms and lowering of the threshold for intoxicating or hangover effects are also due, not to interference with the normal metabolism of alcohol, but to a specific reversible hypersensitivity of cerebral and other target cells.

Although radiation apparently has to be directed to a particular neoplastic focus in order to abolish systemic or local symptoms and induce a shift, the comparatively low dose needed—and the absence of any correlation with tumour cell type or radio-sensitivity—may mean that the effect is on tumour stromal cells rather than on the neoplastic cells themselves (Brewin, 1966). Similar small doses of local radiation, insufficient to eradicate the disease, may produce complete and often lasting remissions in thrombocytopenia associated with haemangioma (Gilon *et al.*, 1959 ; Benninghoff, 1965), pulmonary osteoarthropathy associated with lung carcinoma (Paterson, 1927), and in generalised pruritus due to Hodgkin's disease.

Onset after treatment is another feature of alcohol intolerance that has been reported occasionally with other neoplastic syndromes, including neuromyopathy after removal of lung cancer (Brain, 1963) ; pulmonary osteoarthropathy after radiation of lung cancer (Shapiro, 1956) ; and various auto-immune complications, such as haemolytic anaemia, after radiation or cytotoxic therapy for lymphosarcoma or chronic lymhpatic leukaemia (Lewis *et al.*, 1966). Cyclic uptake of phosphorous by a tumour may be induced by small doses of radiation (Hale, 1966, personal communication).

It looks as if the new systemic effects of a shift may be of two kinds, the first arising from an untreated focus of disease, as when alcohol pain shifts to a new site; the second a transient effect, occurring either with radiotherapy—when no disease exists outside the area being radiated—or after drugs. Perhaps the second type is due to liberation of some substance into the blood stream at the time of treatment. This might occur through breakdown of sensitive tumour stromal cells. Since transient new effects have been seen, not only during treatment, but when alcohol is not taken until some weeks later, it seems possible that this hypothetical substance may persist until neutralised in some way by the taking of alcohol.

Whereas alcohol shift is readily apparent when pain or other local symptom appears at a new site, it is clear that in the case of systemic symptoms it may be difficult or impossible to know that a shift has occurred. No case has been seen where radiation has clearly failed to abolish a local effect ; and it may be that, in some of the cases where systemic intolerance has apparently not responded to radiation, there has in fact been a shift to a new site of origin.

The systemic type of alcohol symptom has been more common in carcinoma, the local type in Hodgkin's disease ; but to what extent this can be explained by the different incidence of multifocal disease is not clear.

The tender nodes that have appeared at new sites of alcohol pain after a shift must presumably have been due to one of three causes : (1) they were not really neoplastic at all (although all were regarded as such and there is no record of spontaneous regression), (2) they were due to rapid acceleration of neoplastic growth, or (3) they were caused by some sudden " allergic " or " inflammatory " change in sub-clinical foci of disease. Present evidence strongly favours the last possibility, but does not completely exclude the other two. Perhaps there might even be some relevance here to animal experiments, some conducted as long ago as 1910, in which complete or partial removal of a primary growth has been followed by apparent enhanced growth of metastases (Marie and Clunet, 1910 ; Schatten, 1958).

How common is alcohol shift? In several neoplastic disorders quite a high incidence of intolerance is found among regular drinkers ; probably this is only because of their greater chance of experiencing it and because of the difficulty of assessing the story of a patient who drinks only occasionally (Brewin, 1966). This suggests that if all patients took alcohol regularly a considerably higher incidence of intolerance might be revealed. Secondly, if all those seen with intolerance had taken alcohol before, during, and after treatment many more cases of shift than the 40 reported here would presumably have been seen. It may be, therefore, that alcohol shift is not just an interesting medical curiosity, but a clue to something more fundamental that is happening not uncommonly in neoplastic disease.

Finally, if alcohol intolerance is associated with secretion of some unknown substance, then could the stimulation of secretion in one region, immediately following its suppression in another, be due to some central influence sensitive to " feed back " changes? Whatever the mechanism, there seems little doubt that a focus of neoplastic disease may begin to show alcohol intolerance for the first time solely because another focus at some distant site has received a modest dose of radiation.

SUMMARY

In Hodgkin's disease, carcinoma of cervix, and other neoplasms, intolerance to alcohol may present in a variety of ways, of which alcohol pain is only one. The term alcohol shift is suggested for the ceasing of one kind of intolerance, usually as a result of local radiotherapy, and its immediate replacement by another. This has been seen 40 times in 33 patients. The new effect may be alcohol pain at a focus of disease which has not previously given rise to this symptom, or it may be systemic alcohol symptoms, such as abnormal vomiting, headache, or flushing, never before experienced. On a further 22 occasions similar alcohol effects have occurred for the first time during or immediately after the treatment of patients who had been tolerating alcohol normally when they had last taken it.

Regardless of dissemination of the disease only one focus normally gives rise to alcohol intolerance, but during a shift multiple alcohol pains may occur, indicating areas of involvement before they are otherwise detectable. In Hodgkin's disease tender nodes may appear at a new site of alcohol pain immediately after the shift.

Alcohol Dysphagia is the term suggested when the oro-pharynx or oesophagus or both, though showing no evidence of disease, give rise to abnormal symptoms, usually a burning sensation, while alcohol is being swallowed. This symptom, which has been observed in 21 patients with various neoplasms, may disappear when radiotherapy is given to a distant neoplastic focus, for example a carcinoma of cervix. Like other forms of systemic alcohol intolerance its onset may be long before diagnosis, or it may appear for the first time much later, sometimes as a transient effect after treatment or during an alcohol shift.

The possible significance of these clinical observations is discussed.

I am indebted to many colleagues, not only in Glasgow, but at Guy's Hospital (where my interest in alcohol pain began in 1953), Westminster Hospital, and the Hamilton clinic of the Ontario Cancer Foundation. I owe a special debt to all those patients with alcohol intolerance who agreed to risk, or to suffer, transient unpleasant effects, in the hope that something might be learned that would help others.

### REFERENCES

ALEXANDER, D. A.—(1953) *Br. med. J.*, ii, 1376.
BENNINGHOFF, D. L.—(1965) *Br. med. J.*, i, 1437.
BICHEL, J.—(1959) *Acta med. scand.*, **164**, 105.
BRAIN, R.—(1963) *Lancet*, i, 179.
BREWIN, T. B.—(1966) *Br. med. J.*, ii, 437.
CONN, H. O.—(1957) *A.M.A. Archs internal Med.*, **100**, 241.
GILON, E., RAMOT, B. AND SHEBA, C. (1959) *Blood*, **14**, 74.
HEALY, J. B.—(1959) *Lancet*, ii, 296.
HOSTER, H. A.—(1950) *Am. J. Roentg.*, **64**, 913.
JAMES, A. H.—(1960) *Q. Jl Med.*, **29**, 47.
JAMES, A. H., HARLEY, H. R., HORTON, E. H. AND STORRING, F. K.—(1957) *Lancet*, i, 299.
LEWIS, F. B., SCHWARTZ, R. S. AND DAMESHEK, W.—(1966) *Clin. exp. Immunol.*, **1**, 3.
MARIE, P. AND CLUNET, J.—(1910) *Bull. Ass. fr. Étude Cancer*, **3**, 19.
PATERSON, R. S.—(1927) *Br. J. Radiol.*, **32**, 435.
SCHATTEN, W. E.—(1958) *Cancer, N.Y.*, **11**, 455.
SHAPIRO, M.—(1956) *A.M.A. Archs internal Med.*, **98**, 700.
WANKA, J.—(1965) *Br. med. J.*, ii, 88.
ZANES, R. P.—(1950) in discussion of Hoster (1950).